# The Relationship Between Body Dysmorphic Disorder and Orthodontic Treatment Need: A Systematic Review

**DOI:** 10.3390/jpm16050271

**Published:** 2026-05-18

**Authors:** Theoklitos Tsaprazlis, Konstantinos Lappas, Miltiadis A. Makrygiannakis, Heleni Vastardis, Eleftherios G. Kaklamanos

**Affiliations:** 1 School of Dentistry, National and Kapodistrian University of Athens, 11527 Athens, Greece; theoklitostsap13@yahoo.gr (T.T.); konslapp@yahoo.gr (K.L.);; 2 School of Dentistry, European University Cyprus, Nicosia 2404, Cyprus; 3 School of Dentistry, Aristotle University of Thessaloniki, 54124 Thessaloniki, Greece; 4 Hamdan Bin Mohammed College of Dental Medicine (HBMCDM), Mohammed Bin Rashid University of Medicine and Health Sciences (MBRU), Dubai P.O. Box 505055, United Arab Emirates

**Keywords:** body dysmorphic disorder, orthodontics, orthodontic indices, IOTN, ICON, systematic review

## Abstract

**Background:** Body Dysmorphic Disorder (BDD) is characterized by an intense preoccupation with perceived flaws in physical appearance, which can influence choices related to aesthetically driven healthcare. In orthodontics, this may cause a mismatch between a person’s subjective concern about their appearance and the treatment need determined by established indices. Therefore, orthodontic treatment indices are crucial to ensure that interventions are clinically justified rather than primarily motivated by disproportionate appearance-related distress. **Objective:** To systematically review and appraise the existing evidence on the connection between BDD and orthodontic treatment need as assessed by established indices. **Materials and Methods:** A systematic search of five electronic databases was conducted for studies published up to March 2026 that examined the association between BDD and orthodontic treatment need. Eligible studies included individuals undergoing orthodontic treatment or seeking orthodontic care, in whom BDD was evaluated using validated instruments and treatment need was assessed using established orthodontic indices. Risk of bias was assessed using the ROBINS-E tool. **Results:** A total of 2743 records were identified, and four observational studies met the inclusion criteria. Due to heterogeneity in study design, assessment methods and outcomes, findings were synthesized narratively. Orthodontic treatment need was assessed using the Dental Health Component of the Index of Orthodontic Treatment Need (IOTN-DHC), the Aesthetic Component of the Index of Orthodontic Treatment Need (IOTN-AC), and the Index of Complexity, Outcome and Need (ICON). Two studies using IOTN-DHC reported a negative association between BDD and orthodontic treatment need, whereas studies using IOTN-AC and ICON found no significant relationship. Associations with sex, age, education, depression, and anxiety were inconsistent across studies. **Conclusions:** Current evidence suggests an inconsistent relationship between Body Dysmorphic Disorder and orthodontic treatment need, highlighting the relevance of personalized assessment in orthodontic decision-making. Given the limited number of studies and the high risk of bias, the findings should be considered preliminary, and further standardized studies are needed to clarify this association.

## 1. Introduction

Body Dysmorphic Disorder (BDD) is a mental health condition characterized by persistent preoccupation with perceived appearance flaws that are slight or not apparent to others [1]. This preoccupation is often accompanied by repetitive acts, including mirror checking and repeated reassurance seeking, leading to distress and functional impairment [2]. Located within obsessive–compulsive and related disorders, BDD involves compulsive behaviors related to body image [3]. It can occur in any sex or age group, but it most often appears during adolescence when concerns about body image become more intense [4].

Despite uncertainty regarding its exact prevalence, BDD is associated with considerable distress, social dysfunction, and mood disturbance, emphasizing the importance of early recognition and clinical awareness [5,6,7]. Adverse early experiences, such as maltreatment or appearance-related teasing, increase vulnerability and reinforce negative perceptions of appearance [6,8,9]. Moreover, broader sociocultural pressures, including exposure to idealized beauty standards, further shape body image dissatisfaction [10].

Given that Body Dysmorphic Disorder is often underrecognized, its management requires an integrated psychological, pharmacological, and psychiatric approach [11,12]. Cognitive Behavioral Therapy (CBT) is generally regarded as the preferred initial treatment approach, aiming to restructure distorted appearance-related beliefs, reduce anxiety through exposure techniques, and prevent compulsive behaviors [8,13,14].

BDD frequently presents in patients seeking medical and cosmetic treatments, where appearance plays a crucial role [15,16,17]. Similar manifestations are also observed in dental and maxillofacial disciplines, particularly among orthodontic and maxillofacial surgery patients who exhibit excessive concern with facial symmetry or minor dental imperfections, even when clinically evident abnormalities are minimal [18,19,20].

Since concerns about distorted appearance may lead individuals with BDD to seek orthodontic treatment, clinicians should adhere to standardized clinical criteria to prevent decisions based solely on subjective dissatisfaction [1]. In this context, orthodontic treatment need indices have been developed to ensure consistent prioritization and to reduce the inherent subjectivity associated with evaluating malocclusion severity [21,22]. The Index of Orthodontic Treatment Need (IOTN) classifies need using dental health criteria and an aesthetic scale that identifies patients most likely to benefit from treatment [21]. The Index of Complexity, Outcome and Need (ICON) provides a unified score incorporating treatment need, case complexity, and outcome, validated against expert orthodontic judgments [22]. The Peer Assessment Rating (PAR) index measures deviation from ideal occlusion and is widely used to quantify improvement following therapy [23]. The Dental Aesthetic Index (DAI) integrates occlusal traits with their psychosocial impact, supporting population-level evaluation of aesthetic burden and treatment need [24]. By grounding decisions in such indices, orthodontists can differentiate true indications from appearance-driven requests, which is especially important when treating patients with BDD [25].

Although recent systematic reviews have examined Body Dysmorphic Disorder in orthodontic and orthognathic contexts [15,26], the specific relationship between BDD and orthodontic treatment need assessed with validated orthodontic indices has not yet been explored. This evidence gap is clinically relevant because index-based assessment may help distinguish standardized treatment need from subjective treatment demand alone. Personalized management of Body Dysmorphic Disorder should recognize that patients may differ in symptom severity, insight, psychiatric comorbidities, functional impairment, and readiness for psychological treatment [27]. Therefore, this systematic review aimed to summarize and critically appraise the available evidence on the relationship between body dysmorphic disorder and the need for orthodontic treatment, with the goal of supporting clinicians in the identification and management of patients with BDD.

## 2. Materials and Methods

### 2.1. Protocol and Registration

This systematic review was conducted following the methodological guidelines of the Cochrane Handbook for Systematic Reviews of Interventions [28]. This review was reported in accordance with the PRISMA statement (Preferred Reporting Items for Systematic Reviews and Meta-Analysis) [29]. The PSISMA checklist can be found in Supplementary Material File S1. The protocol of the review was registered in Open Science Framework (osf.io) (https://osf.io/r2eyu/overview) (accessed on 12 May 2026). The OSF, ID: 10.17605/OSF.IO/R2EYU.

### 2.2. Eligibility Criteria

The focused research question of this systematic review was: “What is the relationship between the Body Dysmorphic Disorder and orthodontic treatment need (assessed with established indices) in individuals seeking or undergoing orthodontic treatment?” We also investigated the prevalence of BDD in the included studies and other potentially associated factors that were examined.

The eligibility criteria for this review followed the Population, Exposure, Comparison, Outcome, and Study Design (PECOS) framework (Table 1).

Specifically, we included studies involving individuals with malocclusion (exposure) of any age who were either seeking orthodontic assessment or undergoing orthodontic treatment for the correction of their malocclusion and whose orthodontic treatment need had been assessed with an established index indicating orthodontic treatment need. The primary outcome of interest was the relationship between the BDD and orthodontic treatment need, assessed with orthodontic treatment need indices. Secondary outcomes included the prevalence of BDD and the presence of other potentially associated factors, such as age, sex, education level, marital status, income, depression, anxiety, type of malocclusion, and previous orthodontic consultation. Studies were eligible regardless of whether they included a comparator group. Systematic reviews, animal studies, and case reports were excluded. No language restrictions were applied.

### 2.3. Information Sources and Search Strategy

A systematic search was conducted across five databases (Medline (PubMed), CENTRAL (Cochrane Library; includes records from Embase, CINAHL, ClinicalTrials.gov, WHO’s ICTRP, KoreaMed, Cochrane Review Groups’ Specialized Registers, and records identified by hand-searching), Scopus, Web of Knowledge (including Web of Science Core Collection, KCI Korean Journal Database, Russian Science Citation Index, SciELO Citation Index, and Zoological Record), and ProQuest Dissertation and Theses (ProQuest)) to identify potentially eligible studies up to March 2026. A broad set of relevant terms was used in the search strategy to ensure the identification of all potentially eligible studies. The search strategy was designed for PubMed and modified for the other databases accordingly (Table 2).

### 2.4. Study Selection, Data Collection, and Data Items

All retrieved records were independently screened by two reviewers (TT and KL). In cases of unclear abstracts, their full text was assessed. Any disagreements were resolved through discussion with additional co-authors (MAM, HV, and EGK) until consensus was reached.

Data extraction was also performed independently by the same reviewers using a predefined form developed for this review. The extracted data included bibliographic information (authors and year of publication), study design, and participant characteristics (sample size, age range, sex distribution, and recruitment setting). Information on the orthodontic treatment need index and the assessment method was also collected, along with the tools used to evaluate Body Dysmorphic Disorder (BDD) and the reported prevalence of BDD. Finally, data on the association between orthodontic treatment need and BDD, as well as other relevant factors related to BDD, were recorded.

### 2.5. Risk of Bias Assessment

The risk of bias for outcomes relevant to the review question was evaluated using the Risk Of Bias In Non-randomized Studies of Exposures (ROBINS-E) tool [30]. The assessment was conducted independently by two reviewers (TT and KL). The ROBINS-E tool considers multiple domains of potential bias, including confounding, exposure measurement, participant selection, post-exposure interventions, missing data, outcome measurement, and selective reporting. Each domain was rated as low risk, some concerns, high risk, or very high risk. An overall risk-of-bias judgment was assigned to each study based on the highest level of risk identified across the assessed domains. Any disagreements were resolved through discussion with additional authors (MAM and EGK) until consensus was reached. The results were visualized using the robvis tool [31].

### 2.6. Data Synthesis, Risk of Bias Across Studies, and Additional Analyses

A quantitative synthesis (meta-analysis) was planned if sufficient homogeneity in study design, outcome measures, and reporting was identified among the included studies. In cases of significant clinical or methodological heterogeneity, a qualitative synthesis of the evidence was pre-specified. Where applicable, assessment of risk of bias across studies, including evaluation of publication bias or small-study effects, was planned.

## 3. Results

### 3.1. Study Selection

The database search yielded a total of 2743 records, including 2701 records identified through electronic databases and 42 records identified via registers. Prior to screening, 90 duplicate records were removed, and a further 1046 records were excluded by automation tools. The remaining 1607 records were screened based on titles and abstracts, leading to the exclusion of 1590 records that did not meet the eligibility criteria.

**Figure 1:**
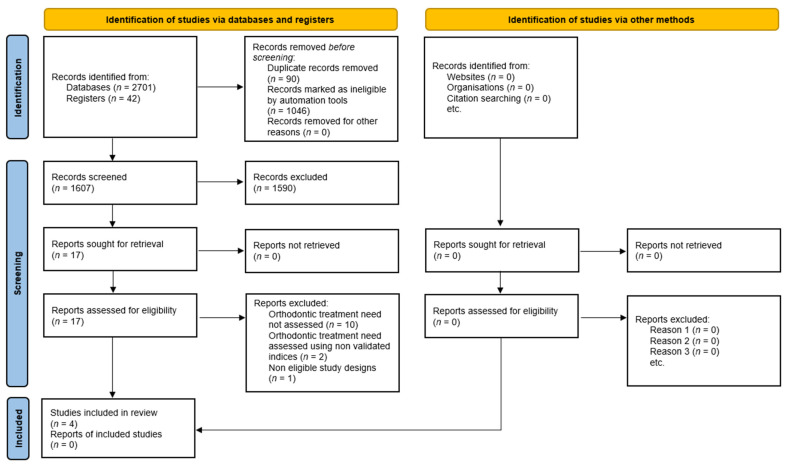
PRISMA 2020 diagram of the included studies.

Seventeen full-text articles were subsequently retrieved and assessed for eligibility. Of these, 13 studies were excluded following full-text review, primarily because orthodontic treatment need was not assessed (*n* = 10), orthodontic treatment need was evaluated using non-validated indices (*n* = 2), or the study design was not eligible (*n* = 1) [18,19,20,32,33,34,35,36,37,38,39,40,41]. Ultimately, four studies fulfilled all inclusion criteria and were included in the qualitative synthesis. The study selection process is illustrated in the PRISMA 2020 flow diagram (Figure 1).

### 3.2. Study Characteristics

The characteristics of the included studies are presented in Table 3.

A total of four observational studies were included in the qualitative synthesis, all employing a cross-sectional design [25,42,43,44]. Three studies were conducted in Iran [25,42,43], and one in Turkey [44]. The studies were published between 2023 and 2026 [25,42,43,44]. The total sample comprised 1722 participants, including 1111 orthodontic patients, 404 patients interested in receiving orthodontic consultation, and 207 general dental patients. All studies used convenience sampling, with sample sizes ranging from 205 to 699 participants. Study populations included adolescents [44], adults [42,43], and mixed samples of both adolescents and adults [25] with females representing the majority of participants in all studies. In fact, the study by Talaei et al. (2025) had only female participants [42].

**Table 3:** Characteristics of the included studies.

Authors, Year and Study Design	Population Characteristics	BDD Assessment Tool	BDD Prevalence	Orthodontic Need Index	BDD in Relation to Orthodontic Need	Other Potentially Associated Factors	Critical Evaluation
**Shiazi et al., 2025 [25]** **Cross-sectional**	404 orthodontic candidates >18 years 82.9% female 17.1% male Dental clinics in Qazvin, Iran	Body Dysmorphic Meta-Cognition Questionnaire (BDMCQ)	56.4% of females have BDD 44.9% of males have BDD 54.5% of adults seeking orthodontic treatment have BDD	IOTN-DHC	Negative relation: Inverse pattern mainly between severe BDD and IOTN grade for Grades 1–3 *p* < 0.05	Age > 41 years (*p* < 0.05) Education: Master’s degree or higher (*p* < 0.05) Sex, marital status and religiousness not significant	BDD–orthodontic treatment need: Negative association BDD associated with age and education
**Sobouti et al., 2023 [43]** **Cross-sectional**	699 orthodontic patients 13–39 years 68.3% female 31.7% male Private clinics in Babol and Sari, Iran	Body Dysmorphic Disorder Yale–Brown Obsessive Compulsive Scale (BDD-YBOCS; 3-item and 12-item versions)	17.0% (3-item BDD-YBOCS) 2.9% (12-item)	IOTN-DHC	Negative: Higher BDD in lower IOTN scores (*p* < 0.05)	Female sex (*p* < 0.001) Married status (*p* = 0.022) Previous orthodontic visit (*p* < 0.001)	BDD–orthodontic treatment need: Negative association BDD associated with female sex, married status, and previous orthodontic consultation
**Talaei et al., 2025 [42]** **Cross-sectional**	414: 207 orthodontic patients, 207 dental patients 100% female 18–35 years Public dental clinic in Mashhad, Iran	Yale–Brown Obsessive–Compulsive Scale Modified for BDD [BDD-YBOCS (12-item)] Psychiatric interview (Diagnostic and Statistical Manual of Mental Disorders, Fifth Edition [DSM-5])	6.28% (orthodontic) 2.89% (controls, *p* < 0.05)	ICON	No correlation between ICON and BDD severity (*p* = 0.804)	Receiving orthodontic procedures (*p* < 0.05) Depression measured with BDI-II (correlated with BDD) (*p* < 0.001) Anxiety measured with BAI (correlated with BDD) (*p* < 0.001) Income (weak negative correlation) (*p* < 0.05) Education and age not significant	BDD–orthodontic treatment need: No significant association BDD associated with depression, anxiety, and income
**Camcı and Geniş, 2026 [44]** **Cross-sectional study**	205 orthodontic patients 12–18 years 69.3% female 30.7% male 12–18 years Department of Orthodontics at the Afyonkarahisar Health Science University, Faculty of Dentistry	Body Dysmorphic Disorder Questionnaire (BDDQ)	69.8%	IOTN-AC	No correlation between IOTN-AC and BDDQ positivity	Depression measured with BDI positively associated with BDD (*p* < 0.001) Weak negative correlation with skeletal malocclusion type (*p* < 0.05) Sex, age, anterior crowding not significant	BDD–orthodontic treatment need: No significant association BDD associated with depression; weak negative association with skeletal malocclusion type

BAI: Beck Anxiety Inventory; BDD: Body Dysmorphic Disorder; BDI-II: Beck Depression Inventory-Second edition; ICON: Index of Complexity, Outcome and Need; IOTN-DHC: Index of Orthodontic Treatment Need-Dental Health Component; IOTN-AC: Index of Orthodontic Treatment Need-Aesthetic Component.

Body Dysmorphic Disorder was assessed using various psychometric instruments, including the 3- and 12-item versions of the Yale–Brown Obsessive–Compulsive Scale modified for BDD (BDD-YBOCS) [42,43], the Body Dysmorphic Metacognition Questionnaire (BDMCQ) [25], and the Body Dysmorphic Disorder Questionnaire (BDDQ) [44].

Orthodontic treatment need was evaluated using established orthodontic indices, including the Dental Health Component of the Index of Orthodontic Treatment Need (IOTN-DHC) [25,43], the Aesthetic Component of the Index of Orthodontic Treatment Need (IOTN-AC) [44], and the Index of Complexity, Outcome and Need (ICON) [42].

### 3.3. Risk of Bias Within Studies

**Figure 2:**
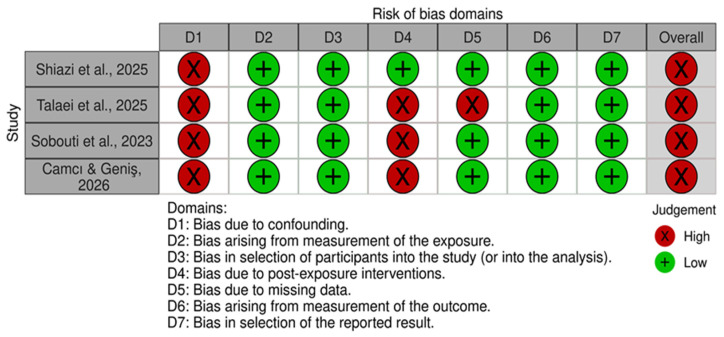
Risk of bias assessment with ROBINS-E tool [25,30,42,43,44].

Figure 2 summarizes the risk of bias assessment. Risk of bias was evaluated using ROBINS-E. All 7 domains for each study were reviewed, and all studies were found to have a high risk of bias, mainly due to confounding. Potential confounding factors that were not consistently controlled for across the included studies included depression, anxiety, socioeconomic status, and previous orthodontic treatment, as well as demographic variables such as age and sex. In addition, none of the included studies reported whether participants with Body Dysmorphic Disorder had sought or received treatment for the BDD, which may represent an additional confounding factor.

### 3.4. Synthesis of Results

A quantitative synthesis (meta-analysis) was not feasible due to substantial methodological heterogeneity across the included studies. This heterogeneity concerned study design, variation in psychometric instruments used to assess body dysmorphic disorder, differences in orthodontic indices applied to evaluate treatment need, and the manner in which outcomes were reported. In addition, the limited number of eligible studies precluded meaningful statistical pooling. Following the recommendations of the Cochrane Handbook, a qualitative (narrative) synthesis was therefore undertaken to appropriately summarize the available evidence [28].

### 3.5. Results of Individual Studies

The lowest BDD prevalence was recorded by Talaei et al., who applied the 12-item version of the Yale–Brown Obsessive–Compulsive Scale modified for BDD (BDD-YBOCS) and reported a prevalence of 6.3% among orthodontic patients [42]. Similarly, when Sobouti et al. used the full 12-item BDD-YBOCS, 2.9% of participants met the diagnostic threshold for BDD, whereas application of the shorter 3-item version resulted in a substantially higher prevalence of 17% [43]. In contrast, Shiazi et al., employing the Body Dysmorphic Metacognition Questionnaire (BDMCQ), reported that more than half of adults seeking orthodontic treatment (54.5%) presented severe BDD symptoms [25]. The highest prevalence was observed in the adolescent sample studied by Camcı and Geniş, where 69.8% of participants screened positive for BDD using the Body Dysmorphic Disorder Questionnaire (BDDQ) [44].

A negative association between BDD and orthodontic treatment need was identified by both Shiazi et al. and Sobouti et al., who assessed treatment need using the Dental Health Component of the Index of Orthodontic Treatment Need (IOTN-DHC) [25,43]. In contrast, Talaei et al., who evaluated orthodontic treatment need using the Index of Complexity, Outcome and Need (ICON), reported no correlation between the severity of malocclusion and BDD [42]. Similarly, Camcı and Geniş, who assessed self-perceived dental esthetics using the Aesthetic Component of the Index of Orthodontic Treatment Need (IOTN-AC), found no association between BDD and orthodontic treatment need [44].

Sociodemographic and psychosocial factors showed variable associations across the included studies. Regarding sex, Sobouti et al. reported higher BDD scores among females, whereas other studies found no significant sex differences [25,43,44]. Overall findings regarding sex were inconsistent across studies. Sobouti et al. observed a higher prevalence of BDD among married participants, whereas other studies found no significant associations [25,42,43]. With respect to age, Shiazi et al. reported increased BDD severity among participants older than 41 years, whereas other studies found no significant age-related associations [25,42,43]. Higher educational attainment was associated with increased BDD severity in the study by Shiazi et al., particularly among participants with postgraduate education [25]. Additionally, Talaei et al. reported a weak negative association between income and BDD severity, suggesting higher BDD scores among individuals with lower income levels [42]. Sobouti et al. also reported a significant association between BDD and previous orthodontic consultation [43]. Talaei et al. reported significant positive correlations between BDD severity and both anxiety and depression scores, while Camcı and Geniş also identified a positive association between BDD and depressive symptoms [42,44]. Findings across studies consistently indicate a positive association between BDD and psychological factors. Additionally, Camcı and Geniş reported a weak negative association between BDD and skeletal malocclusion type [44].

## 4. Discussion

Body Dysmorphic Disorder involves marked distress related to perceived flaws in appearance, typically accompanied by repetitive appearance-related behaviors and significant functional impairment [45,46,47]. Its relevance extends beyond psychiatry, as affected individuals often seek appearance-enhancing interventions in aesthetic-oriented specialties, including dentistry and orthodontics, even when clinically evident abnormalities are absent or minimal [10,19,48].

Evidence from orthodontic settings suggests that some treatment-seeking patients may screen positive for BDD, underscoring the need for clinician awareness and careful assessment when appearance-related concerns appear disproportionate to clinical findings [18]. In orthodontic patients with possible BDD, an important clinical challenge is the potential mismatch between subjective distress about appearance and the assessed severity of malocclusion. In such cases, established orthodontic indices are especially valuable because they provide a more standardized basis for treatment planning [43].

To support the structured assessment of malocclusion and orthodontic treatment need, several indices have been developed. The Peer Assessment Rating (PAR) index mainly measures deviation from ideal occlusion and treatment change, while the Dental Aesthetic Index (DAI) combines occlusal traits into a single score, with emphasis on aesthetic and socially handicapping malocclusion [23,24]. In contrast, the Index of Orthodontic Treatment Need (IOTN) and the Index of Complexity, Outcome, and Need (ICON) are more directly designed to evaluate and prioritize orthodontic treatment requirements [21,22]. In the included studies, orthodontic treatment need was assessed using IOTN-DHC, IOTN-AC, and ICON [25,42,43,44]. These indices support a more structured evaluation and reduce reliance on subjective judgment alone [21,22,49]. However, because their dental health, aesthetic, and complexity-related components capture different dimensions of treatment need, direct comparability across studies may be limited [49,50,51].

Although orthodontic indices provide a standardized approach to treatment-need assessment, orthodontic diagnosis remains broader and should include functional aspects, such as mastication, breathing-related considerations, and functional occlusion, as well as skeletal relationships and growth considerations [52,53]. Soft-tissue analysis and facial aesthetics are also central to comprehensive orthodontic treatment planning, since treatment goals are influenced not only by dental and skeletal findings but also by the soft tissues of the face [52,54]. Contemporary digital and three-dimensional diagnostic approaches may further support this broader assessment by improving the evaluation of facial soft tissues, occlusal relationships, and treatment planning, while remaining adjunctive to comprehensive clinical judgment [53,55]. Therefore, in patients with suspected Body Dysmorphic Disorder, index-based findings should be interpreted together with the interaction between clinically assessed findings and patient perception, including expectations, satisfaction, and aesthetic self-perception [52,56].

Body Dysmorphic Disorder may be assessed with self-report screening instruments and clinician-administered severity scales, depending on whether the aim is initial screening or symptom-severity assessment [45]. Across the included studies, BDD was assessed using the 3- and 12-item versions of the BDD-YBOCS, the BDMCQ, and the 7-item BDDQ, while one study also incorporated psychiatric interview for diagnostic confirmation [25,42,43,44].

The BDD-YBOCS is useful because it provides a validated clinician-administered assessment of current Body Dysmorphic Disorder symptom severity; however, direct comparison across studies is limited when different formats of the scale are used, as the 3-item and 12-item versions may yield markedly different prevalence estimates [43,45]. The BDMCQ could be valuable for assessing the metacognitive aspects of body-image disturbance and has established reliability and validity. However, it is less directly comparable with diagnostic or symptom-severity tools because it was designed to measure metacognitive distortions rather than to diagnose BDD [57]. Likewise, the BDDQ serves as a brief self-report screening tool, but it should not be used alone for diagnosis and is best interpreted alongside a clinical interview [45,58].

Regarding the connection between BDD and orthodontic indices, the included studies did not demonstrate a consistent relationship between BDD and orthodontic treatment need [25,42,43,44]. More specifically, the two studies that applied the IOTN-DHC suggested an inverse association, as higher Body Dysmorphic Disorder symptom scores were observed among participants with little or no index-based orthodontic treatment need [25,43]. Especially in the study by Shiazi et al., an inverse pattern between severe BDD and IOTN grade for Grades 1–3 was observed [25]. By contrast, the study that used the IOTN-AC did not identify a meaningful correlation between IOTN scores and positive screening results on the BDDQ [44]. Similarly, the study that applied the ICON reported no correlation between malocclusion severity and BDD scores [42]. This pattern may reflect a discrepancy between subjective appearance-related distress and standardized orthodontic assessment. In individuals with BDD, exaggerated concern over minor or non-existent defects may contribute to treatment seeking despite limited index-based need. Accordingly, the inverse association observed in the IOTN-DHC studies may be interpreted as reflecting perceptual distortion rather than true orthodontic necessity.

Beyond the orthodontic indices themselves, individual malocclusion characteristics were examined separately in two included studies, yielding partly divergent findings [42,44]. In that respect, Talaei et al. reported no correlation between the severity of malocclusion and BDD scores [42]. By contrast, Camcı and Geniş found a weak but statistically significant negative correlation between malocclusion type and BDDQ positivity, while no meaningful correlation was identified for upper or lower anterior crowding [44]. Similar orthodontic evidence outside the included studies also suggests that malocclusion traits may vary between BDD-positive and BDD-negative patients, although this research remains limited and methodologically inconsistent [37].

With regard to the relationship between BDD and depression, the included studies that assessed depressive symptoms consistently pointed to a positive link between BDD-related measures and greater depressive burden [42,44]. More specifically, Talaei et al. found that higher BDD symptom scores were significantly related to higher levels of depression [42]. In the same direction, Camcı and Geniş identified a moderate positive correlation between BDD symptoms and depression, indicating greater depressive burden among participants with positive BDD screening results [44]. In addition, anxiety was examined in one included study only, where it was also positively related to BDD severity [42]. Overall, these findings are in agreement with the broader BDD literature, which identifies depressive and anxiety disorders among the most common psychiatric comorbidities of BDD [45].

Age was another factor examined in the included studies, although the overall findings were predominantly non-significant, with only one study reporting a significant positive association with BDD-related outcomes [25,42,43,44]. These mixed findings are broadly in line with the wider literature, in which age has not emerged as a consistent factor associated with BDD [59].

Of the four studies included, one involved only women and therefore did not allow for sex-based comparisons [42]. Among the remaining three studies, one reported a significant association with BDD, whereas two did not [25,43,44]. More precisely, Sobouti et al. found that BDD was more prevalent or more severe in female participants, suggesting a positive association between female sex and BDD-related outcomes [43]. These contradictory findings are broadly in line with the wider BDD literature, in which no major sex disparity has been consistently observed in adults [45].

Marital status was examined in three included studies, and the results were inconsistent. One study reported a significant association with BDD, while two others did not [25,42,43]. Overall, these mixed findings only partially align with the broader BDD literature, where marital status has been studied less consistently, although existing evidence more often indicates that married status may be less common among individuals with Body Dysmorphic Disorder [59,60].

The role of education level in relation to BDD was explored in three included studies, but only one reported a significant association, whereas the other two did not identify a statistically significant relationship [25,42,43]. Nevertheless, caution is required when interpreting these findings, as the broader BDD literature has not identified education as a consistent factor associated with the disorder and available studies have shown conflicting results [59].

Income was evaluated in only one included study, which reported a weak negative correlation between income and BDD severity [42]. Specifically, Talaei et al. found that lower income levels were associated with higher BDD severity scores [42]. This finding is broadly compatible with the wider BDD literature, in which lower income has also been reported among individuals with BDD [61].

Previous orthodontic consultation emerged as an additional factor only in one included study, where it was associated with BDD-related outcomes [43]. Notably, Sobouti et al. reported that the 3-item BDD classification was associated with previous orthodontic consultation, identifying a relevant correlation with BDD in their sample [43]. This finding is supported by previous orthodontic studies reporting more frequent prior orthodontic consultations among BDD-positive patients, although similar evidence is less clearly established in the broader BDD literature, where treatment-seeking is usually discussed in more general aesthetic terms [18,33,45].

Finally, religion or spirituality was only examined in the study by Shiazi et al., which found no statistically significant link between religious beliefs and the prevalence of BDD [25]. In the broader BDD research, however, some studies have shown greater BDD symptoms among individuals with lower religiousness or poorer religious beliefs and practices [62,63].

The inverse association observed in the IOTN-DHC studies may reflect perceptual distortion and treatment-seeking behavior despite limited index-based treatment need [25,43]. Accordingly, orthodontists should be cautious about basing treatment decisions solely on subjective dissatisfaction when standardized indices indicate minimal need, and may consider brief psychological screening as a supportive step when appearance-related concerns appear disproportionate to clinical findings [64]. In such cases, screening may help identify patients who could benefit from additional psychological or psychiatric evaluation, without replacing orthodontic diagnosis or treatment planning [64,65]. This approach may be particularly relevant in structured dental and orthodontic settings, where training, referral pathways, and access to mental health professionals could support more integrated patient care [66]. Within a personalized orthodontic framework, treatment need should not be determined solely by occlusal indices or clinician-rated malocclusion severity, but should also incorporate patient-specific psychosocial factors, including, expectations, history of repeated consultations, and screening for Body Dysmorphic Disorder [56,58]. This is particularly important because patients with Body Dysmorphic Disorder may present with limited index-based treatment need while experiencing disproportionate distress, and orthodontic treatment alone may not address the underlying psychological burden [27]. In this context, communication about treatment expectations and careful consideration of satisfaction-related psychosocial factors may further support individualized and patient-centered orthodontic care [67].

## 5. Strengths and Limitations

This systematic review has several strengths. It was conducted according to current methodological guidelines, following a predefined and transparent process for identifying eligible studies, selecting records, extracting data, and assessing risk of bias. In addition, it addressed a focused and clinically relevant question by examining the relationship between the Body Dysmorphic Disorder and orthodontic treatment need using established orthodontic indices rather than subjective treatment demand alone. Another strength is that the review did not restrict its synthesis to prevalence estimates, but also considered differences in BDD assessment tools as well as potentially associated demographic and clinical factors, allowing for a broader interpretation of the available orthodontic evidence.

Despite adherence to current guidelines, which is an important strength of this systematic review, several limitations should be acknowledged, mainly due to the characteristics of the included evidence base. The number of eligible studies was small, and the available evidence was methodologically diverse in terms of study design, sample characteristics, orthodontic indices, BDD assessment tools, diagnostic thresholds, and outcome reporting. This diversity prevented meta-analysis and limited direct comparability across findings. In this context, it is noteworthy that the BDDQ is a screening instrument, BDD-YBOCS is a severity scale, BDMCQ measures metacognitive aspects rather than BDD diagnosis itself, and only one study incorporated a psychiatric interview. As these instruments assess distinct constructs, including screening, symptom severity, metacognition, or clinical diagnosis, prevalence estimates and associations across studies are not directly comparable. Additionally, all included studies were observational and clinic-based, with convenience sampling and mostly female samples, which limits generalizability and confines conclusions to associations rather than causality. It is also worth mentioning that generalizability may be further limited by the existing heterogeneity in the samples of the included studies; one study involved patients seeking orthodontic care, two included orthodontic patients, and another included both orthodontic and dental patients. Further limitations of the evidence include incomplete use of confirmatory psychiatric interviews, inconsistent incorporation of the aesthetic or dental component of the index of orthodontic treatment need, and an overall high risk of bias, mainly due to confounding factors. Moreover, none of the included studies reported how many participants with Body Dysmorphic Disorder had sought or received treatment for the disorder itself, which may represent an additional unmeasured confounding factor. Taken together, these issues reduce confidence in the reliability of the conclusions, which should therefore be interpreted as preliminary and hypothesis-generating rather than definitive.

## 6. Recommendations for Further Research

Future research should aim to address the important methodological limitations identified in the current evidence base. First, larger and more representative samples are needed, ideally through multicenter recruitment and consecutive sampling methods, to enhance generalizability beyond single-clinic convenience samples. Greater geographic diversity is also warranted, as most of the currently available evidence originates from only two countries.

Second, future studies should adopt standardized and clinically robust methods for assessing body dysmorphic disorder. The use of different screening and severity instruments across studies substantially limits comparability. Whenever possible, validated screening tools should be supplemented by structured psychiatric interview or clinician-confirmed diagnosis in order to distinguish true BDD from broader body image dissatisfaction or appearance-related concern. Standardization of diagnostic thresholds would also improve the interpretability of prevalence estimates and associations with orthodontic treatment need.

Third, research in this field would benefit from a more consistent approach to the assessment of orthodontic treatment need. Studies should clearly report which orthodontic indices are used and justify their selection. The combined use of indices such as the IOTN-DHC and IOTN-AC, or similarly comprehensive assessment frameworks, may provide a more balanced understanding of the relationship between clinically assessed malocclusion severity, aesthetic concern, and BDD-related symptoms.

In addition, prospective longitudinal studies are needed to clarify the temporal relationship between BDD symptoms and orthodontic treatment seeking, treatment uptake, and treatment outcomes. Existing cross-sectional evidence cannot determine whether BDD predisposes individuals to seek orthodontic care despite limited index-based need, or whether orthodontic concerns themselves intensify pre-existing appearance-related distress. Longitudinal designs could also help determine whether orthodontic treatment improves, worsens, or has no meaningful effect on BDD symptoms over time.

Finally, future studies should also more carefully examine all potential confounding factors, including age, sex, socioeconomic status, marital status, depression, anxiety, prior orthodontic consultation, and previous aesthetic treatment experiences. Research should also distinguish between adolescent and adult populations, as developmental stage may influence both body image vulnerability and motivations for treatment seeking.

## 7. Conclusions

Current evidence suggests an inconsistent relationship between the Body Dysmorphic Disorder and orthodontic treatment need. Specifically, the two studies using IOTN-DHC suggested that Body Dysmorphic Disorder symptoms may be elevated even when this index indicates little or no treatment need, whereas studies using IOTN-AC and ICON found no significant association. A more consistent association appears to exist with depressive burden, whereas demographic and other clinical factors remain less clearly related. This supports the clinical relevance of the Body Dysmorphic Disorder in orthodontic settings. Given the limited number of studies and the high risk of bias, these findings should be considered preliminary and require confirmation in further standardized studies.

## Figures and Tables

**Table 1:** Eligibility criteria.

Domain	Inclusion Criteria	Exclusion Criteria
Participants	Individuals seeking orthodontic assessment or treatment or undergoing orthodontic treatment	Individuals with major psychiatric or neurological conditions
Exposure	Presence of malocclusion, and orthodontic treatment need assessed by an established orthodontic index	
Comparisons	No comparator or if available: either non-orthodontic patients/non-orthodontic candidates or patients without BDD	
Outcomes	Primary: (a) Presence and/or severity of Body Dysmorphic Disorder (BDD), (b) Orthodontic treatment need measured with an index, such as IOTN, ICON, PAR-index, DAI etc. Secondary: BDD prevalence and other potentially associated factors, such as age, sex, education, marital status, depression, etc.	Body Dysmorphic Disorder presence or severity using non-validated or non-standardized instruments Orthodontic treatment need using non-standardized indices
Study design	Randomized Controlled Trials (RCTs), controlled clinical trials (CCTs), observational studies (cohort, case–control) Studies that assessed orthodontic treatment need using standardized and validated indices Studies that evaluated BDD using validated psychometric instruments or structured diagnostic interviews	Systematic reviews and meta-analyses Animal studies Case reports

**Table 2:** Databases searched (up to 21 March 2026), strategies used, and hits per database.

Database [21 March 2026]	Search Strategy	Hits
**PubMed**	(Dysmorph*[tiab] OR “Somatoform Disorders”[Mesh] OR Somatoform Disorder*[tiab] OR Somatization Disorder*[tiab] OR BDD[tiab] OR “Self Concept”[Mesh] OR Body Imag*[tiab] OR Body representation*[tiab] OR Body schem*[tiab] OR Body sham*[tiab] OR Muscle dysmorph*[tiab] OR Imagined ugliness[tiab] OR Bigorexi*[tiab] OR Adonis complex[tiab] OR Body uneasiness[tiab] OR Body dissatisfaction[tiab] OR Body satisfaction[tiab] OR Self-concept[tiab] OR Body concern*[tiab] OR Body anxiet*[tiab] OR Body discrepanc*[tiab] OR Body distortion*[tiab] OR Self-aware*[tiab] OR Self-image[tiab] OR Self-perception[tiab] OR Self-representation[tiab] OR Social anxiet*[tiab]) AND (Orthognathic surg*[tiab] OR “Orthognathic Surgical Procedures”[Mesh] OR Orthognathia[tiab] OR Jaw surg*[tiab] OR “Surgery, Oral”[Mesh] OR Oral surg*[tiab] OR “Oral Surgical Procedures”[Mesh] OR “Malocclusion/surgery”[Mesh] OR Maxillo mandibular surg*[tiab] OR Maxillofacial surg*[tiab] OR Maxillofacial procedure*[tiab] OR Maxillofacial reconstruct*[tiab] OR “Osteotomy, Le Fort”[Mesh] OR Le-fort osteotom*[tiab] OR Lefort osteotom*[tiab] OR Bilateral sagittal split osteotom*[tiab] OR Genioplast*[tiab] OR Chin reposition*[tiab] OR Mandibular osteotom*[tiab] OR Mandibulotom*[tiab] OR Mandibuloplast*[tiab] OR Mandibular reconstruct*[tiab] OR Maxillary osteotom*[tiab] OR Maxilla osteotom*[tiab] OR Mandible osteotom*[tiab] OR Sagittal split ramal osteotom*[tiab] OR Sagittal split ramus osteotom*[tiab] OR Bimaxillary surg*[tiab] OR “Orthodontics”[Mesh] OR Orthodont*[tiab])	**1311**
**Cochrane Central Register of Controlled Trials**	(Dysmorph* OR (Somatoform NEXT Disorder*) OR (Somatization NEXT Disorder*) OR BDD OR (Self Concept) OR (Body NEXT Imag*) OR (Body NEXT representation*) OR (Body NEXT schem*) OR (Body NEXT sham*) OR (Muscle NEXT dysmorph*) OR (Imagined NEXT ugliness) OR (Bigorexi*) OR (Adonis NEXT complex) OR (Body NEXT uneasiness) OR (Body NEXT dissatisfaction) OR (Body NEXT satisfaction) OR (Self-concept) OR (Body NEXT concern*) OR (Body NEXT anxiet*) OR (Body NEXT discrepanc*) OR (Body NEXT distortion*) OR (Self-aware*) OR (Self-image) OR (Self-perception) OR (Self-representation) OR (Social anxiet*)) AND ((Orthognathic NEXT surg*) OR (Orthognathic NEXT Surgical NEXT Procedures) OR Orthognathia OR (Jaw NEXT surg*) OR (Oral NEXT surg*) OR (Oral NEXT Surgical NEXT Procedures) OR (Maxillo NEXT mandibular NEXT surg*) OR (Maxillofacial NEXT surg*) OR (Maxillofacial NEXT procedure*) OR (Maxillofacial NEXT reconstruct*) OR (Le-fort NEXT osteotom*) OR (Lefort NEXT osteotom*) OR (Bilateral NEXT sagittal NEXT split NEXT osteotom*) OR Genioplast* OR (Chin NEXT reposition*) OR (Mandibular NEXT osteotom*) OR Mandibulotom* OR Mandibuloplast* OR (Mandibular NEXT reconstruct*) OR (Maxillary NEXT osteotom*) OR (Maxilla NEXT osteotom*) OR (Mandible NEXT osteotom*) OR (Sagittal NEXT split NEXT ramal NEXT osteotom*) OR (Sagittal NEXT split ramus NEXT osteotom*) OR (Bimaxillary NEXT surg*) OR Orthodont*) in Record Title OR (Dysmorph* OR (Somatoform NEXT Disorder*) OR (Somatization NEXT Disorder*) OR BDD OR (Self Concept) OR (Body NEXT Imag*) OR (Body NEXT representation*) OR (Body NEXT schem*) OR (Body NEXT sham*) OR (Muscle NEXT dysmorph*) OR (Imagined NEXT ugliness) OR (Bigorexi*) OR (Adonis NEXT complex) OR (Body NEXT uneasiness) OR (Body NEXT dissatisfaction) OR (Body NEXT satisfaction) OR (Self-concept) OR (Body NEXT concern*) OR (Body NEXT anxiet*) OR (Body NEXT discrepanc*) OR (Body NEXT distortion*) OR (Self-aware*) OR (Self-image) OR (Self-perception) OR (Self-representation) OR (Social anxiet*)) AND ((Orthognathic NEXT surg*) OR (Orthognathic NEXT Surgical NEXT Procedures) OR Orthognathia OR (Jaw NEXT surg*) OR (Oral NEXT surg*) OR (Oral NEXT Surgical NEXT Procedures) OR (Maxillo NEXT mandibular NEXT surg*) OR (Maxillofacial NEXT surg*) OR (Maxillofacial NEXT procedure*) OR (Maxillofacial NEXT reconstruct*) OR (Le-fort NEXT osteotom*) OR (Lefort NEXT osteotom*) OR (Bilateral NEXT sagittal NEXT split NEXT osteotom*) OR Genioplast* OR (Chin NEXT reposition*) OR (Mandibular NEXT osteotom*) OR Mandibulotom* OR Mandibuloplast* OR (Mandibular NEXT reconstruct*) OR (Maxillary NEXT osteotom*) OR (Maxilla NEXT osteotom*) OR (Mandible NEXT osteotom*) OR (Sagittal NEXT split NEXT ramal NEXT osteotom*) OR (Sagittal NEXT split ramus NEXT osteotom*) OR (Bimaxillary NEXT surg*) OR Orthodont*) in Abstract—(Word variations have been searched)	**42**
**Scopus**	TITLE-ABS ((Dysmorph* OR “Somatoform NEXT Disorder*” OR “Somatization Disorder*” OR BDD OR “Self Concept” OR “Body Imag*” OR “Body representation*” OR “Body schem*” OR “Body sham*” OR “Muscle dysmorph*” OR “Imagined ugliness” OR “Bigorexi*” OR “Adonis complex” OR “Body uneasiness” OR “Body dissatisfaction” OR “Body satisfaction” OR “Self-concept” OR “Body concern*” OR “Body anxiet*” OR “Body discrepanc*” OR “Body distortion*” OR “Self-aware*” OR “Self-image” OR “Self-perception” OR “Self-representation” OR “Social anxiet*”) AND (“Orthognathic surg*” OR “Orthognathic Surgical Procedures” OR Orthognathia OR “Jaw surg*” OR “Oral surg*” OR “Oral Surgical Procedures” OR “Maxillo mandibular surg*” OR “Maxillofacial surg*” OR “Maxillofacial procedure*” OR “Maxillofacial reconstruct*” OR “Le-fort osteotom*” OR “Lefort osteotom*” OR “Bilateral sagittal split osteotom*” OR “Genioplast*” OR “Chin reposition*” OR “Mandibular osteotom*” OR Mandibulotom* OR Mandibuloplast* OR “Mandibular reconstruct*” OR “Maxillary osteotom*” OR “Maxilla osteotom*” OR “Mandible osteotom*” OR “Sagittal split ramal osteotom*” OR “Sagittal split ramus osteotom*” OR “Bimaxillary surg*” OR Orthodont*))	**675**
**Web of Science™ Core Collection**	(Dysmorph* OR “Somatoform NEXT Disorder*” OR “Somatization Disorder*” OR BDD OR “Self Concept” OR “Body Imag*” OR “Body representation*” OR “Body schem*” OR “Body sham*” OR “Muscle dysmorph*” OR “Imagined ugliness” OR “Bigorexi*” OR “Adonis complex” OR “Body uneasiness” OR “Body dissatisfaction” OR “Body satisfaction” OR “Self-concept” OR “Body concern*” OR “Body anxiet*” OR “Body discrepanc*” OR “Body distortion*” OR “Self-aware*” OR “Self-image” OR “Self-perception” OR “Self-representation” OR “Social anxiet*”) AND (“Orthognathic surg*” OR “Orthognathic Surgical Procedures” OR orthognathic OR “Jaw surg*” OR “Oral surg*” OR “Oral Surgical Procedures” OR “Maxillo mandibular surg*” OR “Maxillofacial surg*” OR “Maxillofacial procedure*” OR “Maxillofacial reconstruct*” OR “Le-fort osteotom*” OR “Lefort osteotom*” OR “Bilateral sagittal split osteotom*” OR “Genioplast*” OR “Chin reposition*” OR “Mandibular osteotom*” OR Mandibulotom* OR Mandibuloplast* OR “Mandibular reconstruct*” OR “Maxillary osteotom*” OR “Maxilla osteotom*” OR “Mandible osteotom*” OR “Sagittal split ramal osteotom*” OR “Sagittal split ramus osteotom*” OR “Bimaxillary surg*” OR Orthodont*) (Title) or (Dysmorph* OR “Somatoform NEXT Disorder*” OR “Somatization Disorder*” OR BDD OR “Self Concept” OR “Body Imag*” OR “Body representation*” OR “Body schem*” OR “Body sham*” OR “Muscle dysmorph*” OR “Imagined ugliness” OR “Bigorexi*” OR “Adonis complex” OR “Body uneasiness” OR “Body dissatisfaction” OR “Body satisfaction” OR “Self-concept” OR “Body concern*” OR “Body anxiet*” OR “Body discrepanc*” OR “Body distortion*” OR “Self-aware*” OR “Self-image” OR “Self-perception” OR “Self-representation” OR “Social anxiet*”) AND (“Orthognathic surg*” OR “Orthognathic Surgical Procedures” OR orthognathic OR “Jaw surg*” OR “Oral surg*” OR “Oral Surgical Procedures” OR “Maxillo mandibular surg*” OR “Maxillofacial surg*” OR “Maxillofacial procedure*” OR “Maxillofacial reconstruct*” OR “Le-fort osteotom*” OR “Lefort osteotom*” OR “Bilateral sagittal split osteotom*” OR “Genioplast*” OR “Chin reposition*” OR “Mandibular osteotom*” OR Mandibulotom* OR Mandibuloplast* OR “Mandibular reconstruct*” OR “Maxillary osteotom*” OR “Maxilla osteotom*” OR “Mandible osteotom*” OR “Sagittal split ramal osteotom*” OR “Sagittal split ramus osteotom*” OR “Bimaxillary surg*” OR Orthodont*) (Abstract) and Preprint Citation Index (Exclude—Database) and Research Commons (Exclude—Database)	**677**
**ProQuest Dissertations and Theses Global**	title((Dysmorph* OR “Somatoform NEXT Disorder*” OR “Somatization Disorder*” OR BDD OR “Self Concept” OR “Body Imag*” OR “Body representation*” OR “Body schem*” OR “Body sham*” OR “Muscle dysmorph*” OR “Imagined ugliness” OR “Bigorexi*” OR “Adonis complex” OR “Body uneasiness” OR “Body dissatisfaction” OR “Body satisfaction” OR “Self-concept” OR “Body concern*” OR “Body anxiet*” OR “Body discrepanc*” OR “Body distortion*” OR “Self-aware*” OR “Self-image” OR “Self-perception” OR “Self-representation” OR “Social anxiet*”) AND (“Orthognathic surg*” OR “Orthognathic Surgical Procedures” OR Orthognathia OR “Jaw surg*” OR “Oral surg*” OR “Oral Surgical Procedures” OR “Maxillo mandibular surg*” OR “Maxillofacial surg*” OR “Maxillofacial procedure*” OR “Maxillofacial reconstruct*” OR “Le-fort osteotom*” OR “Lefort osteotom*” OR “Bilateral sagittal split osteotom*” OR “Genioplast*” OR “Chin reposition*” OR “Mandibular osteotom*” OR Mandibulotom* OR Mandibuloplast* OR “Mandibular reconstruct*” OR “Maxillary osteotom*” OR “Maxilla osteotom*” OR “Mandible osteotom*” OR “Sagittal split ramal osteotom*” OR “Sagittal split ramus osteotom*” OR “Bimaxillary surg*” OR Orthodont*)) OR abstract((Dysmorph* OR “Somatoform NEXT Disorder*” OR “Somatization Disorder*” OR BDD OR “Self Concept” OR “Body Imag*” OR “Body representation*” OR “Body schem*” OR “Body sham*” OR “Muscle dysmorph*” OR “Imagined ugliness” OR “Bigorexi*” OR “Adonis complex” OR “Body uneasiness” OR “Body dissatisfaction” OR “Body satisfaction” OR “Self-concept” OR “Body concern*” OR “Body anxiet*” OR “Body discrepanc*” OR “Body distortion*” OR “Self-aware*” OR “Self-image” OR “Self-perception” OR “Self-representation” OR “Social anxiet*”) AND (“Orthognathic surg*” OR “Orthognathic Surgical Procedures” OR Orthognathia OR “Jaw surg*” OR “Oral surg*” OR “Oral Surgical Procedures” OR “Maxillo mandibular surg*” OR “Maxillofacial surg*” OR “Maxillofacial procedure*” OR “Maxillofacial reconstruct*” OR “Le-fort osteotom*” OR “Lefort osteotom*” OR “Bilateral sagittal split osteotom*” OR “Genioplast*” OR “Chin reposition*” OR “Mandibular osteotom*” OR Mandibulotom* OR Mandibuloplast* OR “Mandibular reconstruct*” OR “Maxillary osteotom*” OR “Maxilla osteotom*” OR “Mandible osteotom*” OR “Sagittal split ramal osteotom*” OR “Sagittal split ramus osteotom*” OR “Bimaxillary surg*” OR Orthodont*)) Filters activated: Full text	**38**

## Data Availability

The data underlying this article derive from those included in the relevant published articles.

## Supplementary Materials

The following supporting information can be downloaded at: https://www.mdpi.com/article/10.3390/jpm16050271/s1, File S1: The PRISMA 2020 checklist.
